# Making great strides in medical laboratory quality

**DOI:** 10.4102/ajlm.v3i2.256

**Published:** 2014-11-03

**Authors:** Michael A. Noble, Robert Martin, Jean-Bosco Ndihokubwayo

**Affiliations:** 1 Program Office for Laboratory Quality Management, University of British Columbia, Canada; 2 Department of Global Health, University of Washington, United States; 3 World Health Organization Regional Office for Africa, Republic of the Congo

Whilst the observation of blood and urine as a commentary of illness and disease can be traced back to Hippocrates as early as 300 BC, the true roots and foundations of the modern medical laboratory as a vital investigation process to better understand pathology and diagnosis were established in the late-19th and early-20th centuries.^1^ Modern day laboratory tests have become the cornerstone for objective data collection to assist, affirm and document diagnoses rather than depending on anecdotal and subjective opinion. Use of highly-crafted optical lenses made microscopic examination of urine, sputum, blood and spinal fluid achievable. Microbiology techniques for blood and sputum culture made the diagnosis of tuberculosis, diphtheria and typhoid both possible and documentable. Examination for bilirubinaemia and abnormal glucose levels also became feasible. The first hospital laboratories were established in London (Guys Hospital) and Baltimore (Johns Hopkins Hospital)^1^ and, by the early 20th century, laboratories began to become a permanent part of the infrastructure of hospitals.

Also in the early part of the 20th century, professional organisations were emerging as self-regulating groups that addressed the competencies of laboratory professionals. However, by the 1940s, FW Sunderman and W Belk were concerned because the evidence and experience in United States laboratories indicated that physicians could not trust laboratory test results. In their key study^2^ they demonstrated that interlaboratory testing of identical samples resulted in considerable variability of test performance and results. This was the origin of proficiency testing as a valuable quality measure that ultimately led to the development of quality control, laboratory inspections and accreditation bodies across the United States, Canada, Europe and Australia.

Fortunately, the foundations of the quality movement had already been developed by giants such as Walter Shewhart, W. Edwards Deming and others, in their studies of statistical design. Medical laboratory quality control and the use of the Levey-Jennings chart^3,4^ were based upon the studies of Shewhart.

Over the next 60 years, many countries saw continued growth and improvement in the medical laboratory based upon the tenets of quality and improvements in accreditation processes. Lessons were learned from a variety of sources. Deming^5^ and Juran^6^ promoted quality measures as they assisted the post-war rebirth of Japanese industry. The military developed tools to ensure that suppliers would deliver high quality, consistent and reliable provisions.^6^ Standards documents, in particular the International Organization for Standardization (ISO) 9001 (Quality Management) and its seed documents (http://www.iso.org/iso/home/store/catalogue_tc/catalogue_detail.htm?csnumber=46486), led the way to internationalising quality as we know it today. These efforts led to further refinements for testing laboratories and medical laboratories alike. Arguably the most significant foundational document for medical laboratory quality improvement in the modern era has been the publication of ISO 15189:2003, 2007 and 2012: *Medical Laboratories – Requirements for Quality and Competence*.^7^

Errors and challenges continued to occur, with perhaps the greatest challenge of the modern era being the recognition that containment of HIV would require even greater strides in healthcare improvement and enhancement of medical laboratory quality.^8^ Great efforts by the US President’s Emergency Plan for AIDS Relief (PEPFAR)^9^ programme, the Bill and Melinda Gates Foundation, the Clinton Health Access Initiative and the World Health Organization (WHO) pointed the way for laboratory improvement in every part of the world. It is through those processes and the active energy of the WHO’s Regional Office for Africa (AFRO) that we see the growth and successes of quality management system programmes such as Stepwise Laboratory Quality Improvement Process Towards Accreditation (SLIPTA) and Strengthening Laboratory Management Toward Accreditation (SLMTA).

Time has demonstrated that the path to improvement is not always a straight line; there are periods of success and periods of fallback. Laboratory improvement must be experienced as a slow and gradual process based upon building a culture of quality, increasing knowledge and a goal of continual improvement. The study of risk management teaches us that we cannot predict all errors; but through the active practice of quality processes we can detect errors earlier and reduce the repetition of the same error time and time again. It is through the persistence of personal interest and action that we sustain quality efforts and have confidence in successful outcomes for the overall process of laboratory investigation.

The authors of the excellent manuscripts in this special issue of the African Journal of Laboratory Medicine, entitled *Transforming the Quality of Laboratory Medicine through the Strengthening Laboratory Management Toward Accreditation Program*, attest to the great strides that are being made through the progressive stepwise approach to adopting and embracing quality measures. But to them we put forward this challenge: whilst it is a great achievement to reach a level of success where the requirements of accreditation are met, the true accomplishment is reaching the point where that level of quality is an everyday practice and expectation, and the laboratory is ‘accreditation-ready’ over and over. When the inevitable slips and mistakes occur in laboratories that are accreditation-ready, the processes of error detection, correction and improvement, and progress back to quality, must occur quickly, smoothly and sustainably.^10^
